# Non-specific skin lesions in angioimmunoblastic T-cell lymphoma lead to diagnosis challenge: a case report and literature review

**DOI:** 10.3389/fonc.2026.1785153

**Published:** 2026-05-14

**Authors:** Zimeng You, Qianqian Wang, Li Xue, Lin Wang, Tingting Wang

**Affiliations:** 1 Department of Dermatovenereology, West China Hospital, Sichuan University, Chengdu, China; 2 Laboratory of Dermatology, Clinical Institute of Inflammation and Immunology (CIII), Frontiers Science Center for Disease-related Molecular Network, West China Hospital, Sichuan University, Chengdu, China

**Keywords:** angioimmunoblastic T-cell lymphoma, clinicopathological correlation, cutaneous manifestations, early diagnosis, pathologic characteristic

## Abstract

**Background:**

Angioimmunoblastic T-cell lymphoma (AITL), a rare and aggressive subtype of T-cell lymphoma, affects the skin in up to 50% of cases. Its protean clinical and histopathological cutaneous manifestations pose a challenge in diagnosis, particularly when skin involvement precedes lymph node biopsy confirmation.

**Case presentation:**

A 63-year-old male initially presented with progressive erythema and nodules, later developing vasculitis-like ulcers and generalized lymphadenopathy. The skin biopsy of erythema demonstrated mild perivascular lymphocytic infiltration around small blood vessels throughout the entire dermis, while the vasculitis-like ulcer biopsy demonstrated perivascular lymphocytic infiltration and small blood vessel hyperplasia. The final diagnosis of AITL was made through lymph node incisional biopsy based on morphology and immunohistochemistry. The patient received chemotherapy with the miniCHOP regimen. The skin lesions improved after treatment, and the patient remained under follow-up.

**Conclusion:**

Cutaneous manifestations of AITL are diverse, non-specific and can precede systemic symptoms. Histopathologically, cytological atypia is rarely reported. Immunohistochemistry for T-helper markers and molecular data is sometimes useful. However, both clinical manifestations and histopathology of skin lesions are often non-specific and diverse, leading to misdiagnosis and treatment delay. Continuous observation is necessary in AITL patients who develop skin lesions prior to lymphadenopathy, while the final diagnosis of AITL is still based on lymph node biopsy. Clinicians should be vigilant regarding the cutaneous manifestations of AITL to make an early and accurate diagnosis.

## 1 Introduction

Angioimmunoblastic T-cell lymphoma (AITL) is a rare and aggressive subtype of mature T-cell lymphoma (MTCL) of follicular T-helper (T_FH_) cell origin, which comprises 15%–20% of MTCLs and 1%–2% of all non-Hodgkin lymphomas. Characteristically, AITL presents an inverse geographical tropism to other subtypes of T-cell lymphomas, being more common in Europe and less prevalent in Asia and North America (1).

Clinically, it is characterized by generalized lymphadenopathy, hepatosplenomegaly, constitutional B symptoms (such as fever and weight loss), hypergammaglobulinemia, and cutaneous manifestations. The latter occur in 40%–50% of cases and include morbilliform eruptions, petechiae, urticaria, purpura, papulonodules, erythroderma, and others (2, 3). Vasculitic ulcers are rarely reported (4). Meanwhile, pruritus is also a common symptom in patients with AITL (2, 3). Skin involvement in AITL is usually diverse and non-specific, may evolve with disease progression, and may sometimes be mistaken for diseases such as viral exanthem, autoimmune diseases, or drug eruptions, especially in the early stage (4, 5). While frequently present at diagnosis, cutaneous lesions can precede systemic lymphoma by up to 2 years (6). The early phase of cutaneous lesions lacks specificity and also eludes identification on pathological grounds alone (7). Therefore, diagnosing AITL without lymphadenopathy is challenging, frequently leading to misdiagnosis and delayed treatment (4, 8).

AITL patients with non-specific skin lesions might be referred to non-hematology clinicians. It is essential for clinicians to recognize the clinical and pathological characteristics of skin involvement in AITL to facilitate early and accurate diagnosis to the greatest extent possible.

Here, we present a case of an AITL patient initially manifesting as erythema and nodules that progressed to vasculitic ulcers—both non-specific findings. The skin lesions preceded lymphadenopathy, complicating early consideration of AITL. This case highlights the diagnostic difficulty of AITL in the absence of lymphadenopathy. Meantime, we summarize the cutaneous manifestations and pathological characteristics of AITL to enhance clinicians’ awareness of this disease.

## 2 Case presentation

**Figure 1:**
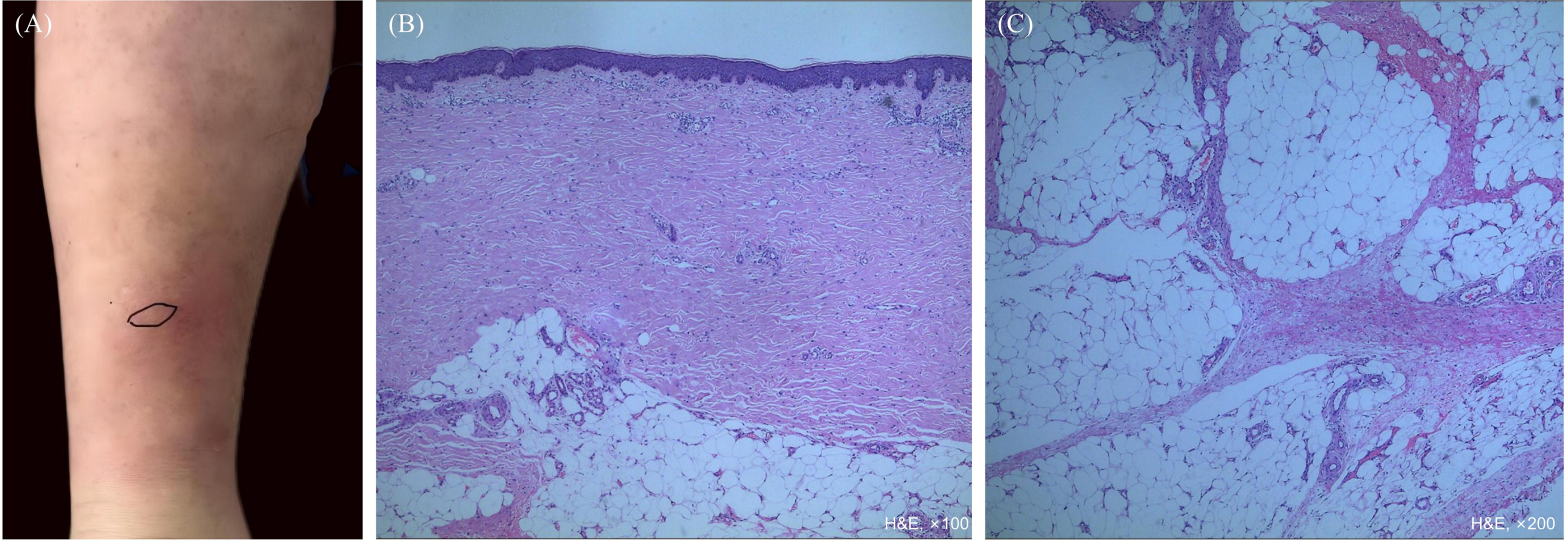
Clinical manifestations and histopathology of erythema. **(A)** Erythema and nodules on the legs; **(B)** Slight acanthosis in the epidermis, with mild perivascular lymphocytic infiltration around small blood vessels throughout the entire dermis; **(C)** The subcutaneous adipose tissue demonstrates widened septa with sparse lymphocytic infiltration, along with sparse lymphocytic infiltration within the fat lobules.

A 63-year-old male was admitted to the hospital with a 1-year history of erythema and nodules on both lower extremities, accompanied by multiple lymphadenopathy over the past 4 months. One year ago, the patient developed unequal-sized erythema and nodules on both legs, and the skin biopsy showed scant perivascular lymphocytic infiltration in the superficial dermis and scattered lymphocytes in the subcutaneous fat layer (**Supplementary Figure 1**). During treatment with compound glycyrrhizin, the lesions recurred and worsened (**Figure 1A**). A repeat biopsy of the erythema lesion was conducted due to poor response to therapy and development of skin lesions, which demonstrated mild perivascular lymphocytic infiltration around small blood vessels throughout the entire dermis and widened septa with sparse lymphocytic infiltration in the subcutaneous fat layer (**Figures 1B, C**). He was diagnosed with nodular erythema and showed partial symptomatic improvement following treatment with compound glycyrrhizin, Haitang mixture (traditional Chinese herbs), and total glucosides of paeony capsules.

Four months ago, he developed lymph node enlargement in the cervical, axillary, and inguinal regions without symptoms, which remained untreated. Meanwhile, he developed herpes zoster and viral encephalitis, which were treated with acyclovir.

**Figure 2:**
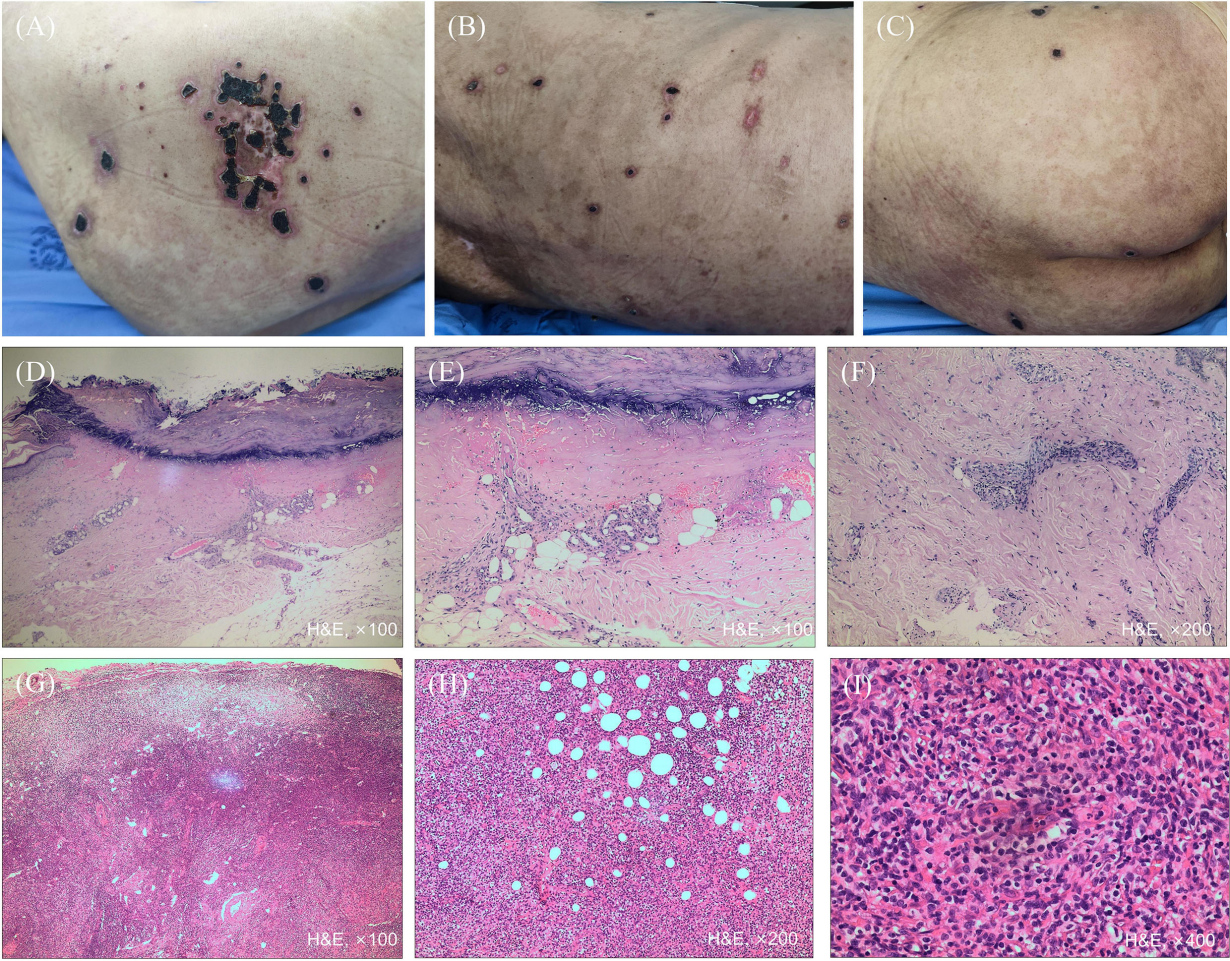
Clinical manifestations and histopathology of vasculitis-like ulcers and inguinal lymph nodes. **(A–C)** Erythema and vasculitis-like ulcers on the trunk and extremities; **(D, E)** Histopathology of the ulcer showed skin ulcer formation with mild irregular hyperplasia of the remaining epidermis, focal liquefactive degeneration of the basal layer, and dermal atrophy beneath the ulcer; **(F)** Histopathology of the ulcer showed collagen fiber hyalinization, small blood vessel hyperplasia, and perivascular infiltration of a small number of lymphocytes in the superficial dermis; **(G)** The lymph node incisional biopsy showed lymphoid hyperplasia, predominantly composed of medium- to large-sized lymphoid cells, accompanied by proliferation of small blood vessels with endothelial cell swelling and clusters of histiocytes; **(H, I)** The lymph node incisional biopsy showed medium- to large-sized lymphoid cells.

One month ago, the skin lesions progressed and worsened significantly, with new-onset bean-to-peanut-sized erythema and painful hemorrhagic bullae that later became vasculitis-like ulcers on the trunk and extremities (**Figures 2A–C**), accompanied by progressive lymphadenopathy. Treatment with prednisone (initially 20 mg/day, tapered to 10 mg/day) failed to achieve significant clinical improvement. The patient developed fever, and ascites occurred after admission.

Laboratory examinations showed mild anemia (103 g/L, normal range: >120g/L), thrombocytopenia (75 × 10^9^/L, normal range: >100 × 10^9^/L), hypoproteinemia (32.8 g/L, normal range: >40 g/L), elevated CRP (51.1 mg/L, normal range: <10 mg/L), PCT (3.6 ng/mL, normal range: <0.046 ng/mL), immunoglobulin E (23,800 IU/L, normal range: <165 IU/L), interleukin-6 (75.6 pg/mL, normal range: <7 pg/mL), ferritin (1,164 ng/mL, normal range: 70 ng/mL–400 ng/mL), interleukin-2 receptor (17,590 U/mL, normal range: 223 U/mL–710 U/mL), and positive Epstein–Barr virus DNA (5.14E + 04 copies/mL, normal range: <50 copies/mL). PET/CT demonstrated multiple lesions in multiple lymph nodes, spleen, and bone marrow, suggesting a hematologic malignancy. Abnormal T lymphocytes (CD4+) of T_FH_ origin were detected in ascites.

As for the bone marrow examination, bone marrow smear demonstrated hypercellularity, with lymphocytes comprising 45% of the cellularity. Approximately 6% of these lymphocytes exhibited significant morphological atypia. The biopsy pathology confirmed hypercellularity with infiltration by a mixed population of T- and B-phenotype lymphocytes.

The newly developed skin lesions differed from the previous erythema. During follow-up, the patient developed an infection and lymphadenopathy. Repeat skin biopsy of vasculitis-like ulcer demonstrated focal liquefactive degeneration of the basal layer, collagen fiber hyalinization, small blood vessel hyperplasia in the superficial dermis, and perivascular infiltration of a small number of lymphocytes (**Figures 2D–F**). An inguinal lymph node biopsy demonstrated lymphoid hyperplasia, predominantly composed of medium- to large-sized lymphoid cells, accompanied by proliferation of small blood vessels with endothelial cell swelling and clusters of histiocytes (**Figures 2G–I**). The lymphoid cells were positive for CD3, CD5, CD4, negative for CD20, CD79a, and CD8, with focal expression of CD10 (**Supplementary Figure 2**). Among lymphocytes, the majority exhibited positivity for CD3, CD5, CD4, CD2, and CD7, while CD20 and CD79a were only expressed in a minority of cells. CD30 is positive in a subset of transformed cells, and CD21 highlights follicular dendritic cell (FDC) networks. Markers including TdT, CD56, Granzyme B, and pan-cytokeratin (CK Pan) are negative. Additionally, partial positivity is observed for CD10, BCL-6, CXCL-13, and PD-1, while CD8 is positive in a minority of cells. *In situ* hybridization demonstrated scattered positivity for EBER. The Ki-67 proliferation index reached 60%–70% (**Supplementary Figure 3**). Molecular pathology studies detected clonal peaks within the target band ranges for both IgH and *TCRG* gene rearrangements. A pathogenic mutation in exon 2 of the *RHOA* gene was observed.

Based on these results, the patient was diagnosed with AITL and received chemotherapy with the miniCHOP regimen (cyclophosphamide, pirarubicin, vincristine, and prednisone) after diagnosis. The skin lesions improved after treatment, and the patient remained under follow-up. Clinical timeline is outlined in **Table 1**.

**Table 1:** Timeline of the case report.

Time point	Event
September 2023	Erythema and nodules on both legs. Skin biopsy: lymphocytic infiltration in the superficial dermis.
November 2023	Recurrence of erythema and nodules. Repeat skin biopsy: mild perivascular lymphocytic infiltration throughout the entire dermis.
May 2024	Multiple lymphadenopathy and development of herpes zoster and viral encephalitis.
August 2024	New-onset erythema and painful hemorrhagic bullae.
September 2024	Vasculitis-like ulcers, with progressive lymphadenopathy. Development of fever and ascites. Repeat skin biopsy: small blood vessels hyperplasia in the superficial dermis and perivascular infiltration of lymphocytes.
Late September 2024	Inguinal lymph nodes biopsy: lymphoid hyperplasia, predominantly composed of medium to large-sized lymphoid cells. Final diagnosis of AITL based on morphology and immunohistochemistry.
October 2024	Chemotherapy with the miniCHOP regimen. The skin lesions improved after two weeks.

## 3 Discussion

AITL derives from the monoclonal proliferation of T_FH_ cells, with a 5-year survival of approximately 30%–33% in most reported patient series (1, 9). Early diagnosis of AITL is challenging; the majority of AITL patients have advanced-stage disease (Ann Arbor III or IV), and rare cases of early-stage disease (I or II) have been reported and constitute less than 10% of clinical presentations (1). Cutaneous manifestations are the most common extranodal expressions in AITL patients, occurring in up to 50% of cases. Reports on the prognostic impact of skin involvement vary, with some suggesting it correlates with shorter survival (9). In China, the skin involvement may be less frequent, reported at 13.1% in one retrospective analysis (10).

Skin lesions are frequently present at diagnosis but can precede systemic symptoms (6). Diagnosing AITL early without lymphadenopathy is difficult, as it typically requires lymph node incisional biopsy (1). Here, we summarize relevant clinical and histopathological data on skin involvement in recent AITL cases in **Table 2** (2–6, 8, 9, 11–23).

**Table 2:** The clinical and histopathological data on skin involvement in AITL cases in the literature.

Case reports		Year	Age/Sex	Skin lesions	Location	Pathology		
						Hematoxylin–eosin staining	Immunohistochemistry	
							Positive	Negative
1	Jacobson et al. (11)	2013	69/F	Pink-red papules	Upper chest	Superficial and deep perivascular and intralymphatic infiltrate of atypical lymphocytes, with scattered mitoses and admixed plasma cells	CD31, CD41, CD51, EBER-ISH	–
2	Kim et al. (8)	2016	48/F	Skin-colored nodules and ulcer later	Both calves	Prominent vascular proliferation surrounded by abundant epitheloid histiocytes and lymphocytes without prominent nuclear atypia in the dermis	CD3, CD68, CD31	S100, CD1a, EBER-ISH.
3	Maher et al. (12)	2016	71/M	Saggy skin	Back, axillae and periorbital region	Sparse perivascular dermal T-cell infiltrate in the superficial and lower dermis.	CD3, CD4, CD10	CD8, EBER-ISH
4	Kang et al. (13)	2016	56/M	Lip edema, red to violaceous plaques	Face, neck, and anterior chest	Dense lympho-histiocytic dermal infiltrate composed of atypical lymphoid cells ranging in cell size	–	
5	Mangana et al. (14)	2017	65/F	Red confluent patches and plaques	Trunk, extremities	Spongiotic dermatitis with perivascular lymphocytic infiltration with eosinophil	–	
6	Liaw T et al. (15)	2017	84/F	Annular erythematous patches	Trunk, extremities	–	–	
7	Hoskins et al. (4)	2019	54/F	Petechia, purpura, necrotic ulcers	Lower extremities	Epidermal necrosis, superficial and deep perivascular infiltrate of lymphocytes, plasma cells and neutrophils, and small to medium vessel vasculitis, with vessel wall necrosis and fibrin deposition in the upper dermis	–	
8			62/F	Edematous plaques	Lower extremities, back	Superficial perivascular inflammatory infiltrate with numerous eosinophils	–	
9	Murao et al. (16)	2019	65/F	Erythematous papules	Face	Dense superficial, deep perivascular, and interstitial infiltration of the dermis. Infiltrates consisted of numerous lymphocytes without nuclear atypia, eosinophils, histiocytes, and some neutrophils	CD3, CD4, CD79a	PD-1, EBER-ISH
10	Wawrzycki et al. (9)	2021	68/M	Erythematous papules, plaques, purpura	Trunk, proximities	Perivascular infiltrates of small and medium sized lymphocytes in dermis	CD3, CD4	–
11	Pesqué et al. (6)	2022	59/F	Pink to red papules	Forehead	Nodular lymphoid infiltrates on the upper and mid dermis. Dermal infiltrates were mainly composed of lymphoid aggregates by a discrete infiltrate composed of small-to-medium lymphocytes.	CD20, IgD, BCL-2, CD23	BCL-6, CD10
						Dermal infiltrates were mainly composed of lymphoid aggregates surrounded by a discrete infiltrate composed of small-to-medium lymphocytes	CD3, CD4, PD1, CXCL-13, EBER-ISH	–
12	Keefe et al. (17)	2022	65/M	Erythema and scale	Face, trunk, extremities	–	–	
13	Kumar et al. (2)	2023	67/M	Papules, nodules, depigmented and hyperpigmented macules	Trunk, extremities	Irregular acanthosis, fused rete ridges with vertically oriented collagen bundles and pigment incontinence	–	
14	Charest et al. (3)	2023	71/M	Erythematous macules, patches, papules, plaques	Trunk, bilateral upper extremities	Mild perivascular lymphocytic infiltrate in the dermis	PD-1, CD3, CD5, BCL-6, EBER-ISH	–
15	De Clippele et al. (18)	2023	50/F	palpebral edema, erythema.	Eyelid, trunk	Moderately dense perivascular lymphoid infiltrate in the dermis and upper hypodermis, composed of atypical small−to−medium cells	CD4, CD4, CD5, CD7, PD-1, BCL-6, CXCL-13, ICOS	EBER-ISH
16	Choo et al. (19)	2023	56/M	Erythematous macules	Trunk, extremities	Mild epidermal intercellular edema and superficial dermal mild perivascular inflammatory infiltrate	–	
17	Kunitomi et al. (5)	2024	74/M	Erythematous urticaria-like rash	Abdomen, extremities	–	–	
18	Hsueh et al. (20)	2025	77/M	Pseudovesicular papuloplaques, purpura	Trunk, extremities	Mild hyperkeratosis, marked papillary edema, and heavy lymphocytic infiltrates beneath the edema	CD4, PD-1, CXCL-13	CD8, CD20, CD30
19	Mathieu et al. (21)	2025	70/F	Indurated eyelid edema	Eyelid	Thin orthokeratotic epidermis and superficially edematous dermis. Perivascular infiltrates consisted of lymphocytes, histiocytes and numerous eosinophils in deep dermis and the subcutaneous tissue. The lymphocytes were small, with irregular, angular nuclear contours.	CD3, CD4, PD-1, CD10, BCL6, CXCL-13, EBER-ISH	–
20	García et al. (22)	2025	62/F	Erythematous macules and papules	Face, trunk and extremities	Superficial dermal lymphohistiocytic infiltrate	PD-1, EBER-ISH	–
				Subcutaneous nodules	Face, preauricular region	Subtle perivascular dermal and subcutaneous infiltration by intermediate-sized lymphoid cells, eosinophils and histiocytes	CD20, CD30, EBER-ISH	–
				Erythema and ulcer	Left forearm	Diffuse infiltration of the dermis and subcutaneous tissue by large lymphoid cells, with extensive perivascular infiltration that also involved basal epidermal cells.	CD3, CD4, PD-1, CD10, CD20, CD30, CXCL-13, EBER-ISH	–
21	Xie et al. (23)	2026	81/M	Erythematous macules, papules	Trunk, extremities	Focal infiltration of atypical tumor cells involving the epidermis and superficial dermis	CD3, CD4, CD5, CD8	–

BCL-6, B-cell lymphoma-6; CXCL13, CXC motif chemokine 13; PD1, programmed cell death protein 1; EBER-ISH, Epstein–Barr virus (EBV) by *in situ* hybridization.

Cutaneous manifestations of AITL are clinically diverse and often non-specific, manifesting as morbilliform eruptions, petechiae, urticaria, purpura, papulonodules, erythroderma, and others (3). In general, skin lesions in AITL can be broadly categorized into three main patterns: (1) macular, (2) papular, and (3) plaque-like/nodular lesions, or mixed features of the above (7, 17). Usually, maculopapular eruptions are the most common clinical presentation, typically seen in early stages, whereas nodular forms tend to occur late in the disease (6). Among the recent cases of cutaneous manifestations in AITL patients (**Table 2**), the macules, papules, and nodules were the most common manifestations, while rare skin lesions include sagging skin, hyperpigmentation, lip and eyelid edema, pseudovesicular papuloplaques, and ulcers. Other researchers classified AITL cutaneous manifestations into two groups: evanescent, pruritic, reticulated erythematous-violaceous rash; and persistent, non-pruritic, erythematous, indurated papules, plaques, or nodules in a retrospective study (24). Nevertheless, not all clinical cases conform to this dichotomy.

The skin manifestations may also evolve with disease progression, yet few studies have documented sequential changes in cutaneous lesions during follow-up. Only García et al. (22) described a patient who developed distinct cutaneous findings (erythema, nodules, and ulcers) over a nearly five-year follow-up period, with the initial clinical manifestation emerging approximately five years before the subsequent two lesions. Our patient initially presented with erythema and nodules, later developing vasculitis-like ulcers—a rarely documented transition which could be easily misdiagnosed as erythema nodosum and vasculitis.

Such non-specific and polymorphic lesions complicate accurate diagnosis, as AITL often mimics benign inflammatory dermatoses (e.g., viral exanthems, autoimmune disorders, or drug eruptions) and malignant skin diseases (such as mycosis fungoides), leading to frequent misdiagnosis or delay. This case again underscores the diagnostic challenges in AITL patients, particularly those without lymphadenopathy.

Due to the diverse dermatological manifestations and diagnostic challenges of AITL, cutaneous biopsies are needed to make an accurate diagnosis. However, the histopathological features of cutaneous involvement remain incompletely characterized. Skin lesions typically exhibit polymorphous features characterized by perivascular infiltrates of small-to-medium-sized T-cells, accompanied by reactive histiocytes, plasma cells, and/or eosinophils. However, characteristic findings in lymph node biopsy of AITL, such as atypical lymphocytes with clear cytoplasm and capillary hyperplasia, were present in only a minority of cutaneous biopsies, emphasizing that the diagnostic criteria for nodal AITL may not be entirely applicable to cutaneous lesions (25).

Botros et al. (7) have revealed four histopathological patterns: (1) mild superficial perivascular infiltrate of lymphocytes and eosinophils with capillary hyperplasia; (2) mild superficial perivascular infiltrate of lymphocytes and eosinophils with capillary hyperplasia and lymphocyte cytological atypia; (3) dense superficial and deep infiltrates of pleomorphic atypical lymphocytes with vascular hyperplasia; and (4) vasculitis without lymphocyte cytological atypia (7). Most reported cases align with these four patterns, though atypical lymphocytes are still uncommon. Rare features such as pigment incontinence are also documented (**Table 2**). Recently, Díaz de la Pinta et al. (24) described a spectrum of infiltration patterns, including perivascular, nodular, granulomatous, panniculitic, vasculitic, and epidermotropic patterns, which resemble those described by Botros et al. (7). Lee et al. further described periappendageal lymphocytic infiltration (26).

Clinicopathological correlations reveal that perivascular lymphohistiocytic infiltrates and mixed dermal infiltrates correspond clinically to macular/papular eruptions, whereas dense nodular infiltrates align with plaque-like or nodular lesions. Microscopically, a parallel increment in the density of the dermal infiltrate and in the detection of lymphocyte cytological atypia was noted over time (7). Papulonodular lesions mainly consist of atypical lymphocytes with a perivascular or periappendageal pattern (26). While macular/papular eruptions mimic inflammatory processes microscopically, plaque-like/nodular lesions demonstrate definitive lymphoma features characterized by increased infiltrate density and frequent lymphocyte cytological atypia. However, the maculopapular lesions in AITL still elude identification on pathological grounds alone, even in nodular lesions of AITL, it can also be difficult to establish a histopathological diagnosis (7).

In the case we presented, the first and second skin biopsies of erythema showed varying degrees of dermal perivascular lymphocytic infiltration without cytological atypia. The subsequent vasculitis-like ulcer (post-lymphadenopathy) revealed superficial dermal small-vessel hyperplasia and atypia-free perivascular lymphocytic infiltration. García et al. (22) reported a patient who was ultimately diagnosed with AITL after three sequential skin biopsies for distinct cutaneous manifestations. The first biopsy, obtained from erythematous lesions, revealed superficial dermal perivascular lymphocytic infiltration. Five years later, the second biopsy from newly developed nodules showed dermal infiltration of atypical lymphocytes, and the patient was misdiagnosed with diffuse large B-cell lymphoma. The definitive diagnosis of AITL was eventually established based on the third skin biopsy (from newly developed erythema and ulcers) and lymph node biopsy results. The retrospective study by Díaz de la Pinta et al. (24) also found that distinct clinical and histopathologic patterns could coexist in the same patient over time. These non-specific and diverse clinicopathological findings complicated early suspicion of AITL.

Given the poor treatment response and the recurrent, variable morphology of skin lesions, we performed continuous cutaneous monitoring and repeated skin biopsies targeting lesions with distinct clinical presentations. Similarly, García et al. (22) described a case with evolving skin manifestations during follow-up. Continuous clinical surveillance is necessary and meaningful, because early cutaneous lesions of AITL often lack specific clinical and histopathological features. Repeat skin biopsies, together with lymph node biopsy, are frequently required to achieve a definitive and accurate diagnosis, especially in cases where skin involvement precedes lymphadenopathy.

Therefore, immunohistochemistry for T_FH_ markers is useful in addressing the difficulty in differential diagnosis between reactive skin conditions and cutaneous involvement by AITL. CXC motif chemokine 13 (CXCL13), programmed cell death protein 1 (PD-1), inducible T-cell costimulator, CD10, and B-cell lymphoma 6 protein (BCL-6) currently represent the most useful and robust immunohistochemical markers of T_FH_ cells, which were used in some reported cases (3, 6, 18, 20–22). Cutaneous lesions also demonstrate lower EBV-positive cell prevalence—a potential independent prognostic factor in systemic AITL (25, 26). However, reported cases (**Table 2**) and retrospective analyses (7, 25, 26) show variable expression of these markers. Expression of T_FH_ markers is helpful but should be assessed in the context of a panel of immunohistochemical stains, because T_FH_ markers vary in their individual sensitivity and specificity. Meanwhile, molecular testing plays a critical auxiliary role. *RHOA, TET2*, and *IDH2* mutations have also been detected in cutaneous lesions, with mutation profiles generally consistent between skin and lymph node biopsy specimens (24). Given the non-specific and diverse histology of cutaneous AITL, definitive diagnosis requires clinicopathological correlation (25).

Nonetheless, there are still some clues for clinicians to consider the possibility of AITL. For the early maculopapular phase, in the context of (1) a rash that simulates benign inflammatory dermatoses but proves recalcitrant, (2) constitutional abnormalities and/or persistent lymphadenopathy, and (3) a T_FH_ cell-rich perivascular dermatitis, early cutaneous manifestation of AITL can be suspected (7). Recently, researchers found that molecular data could provide valuable auxiliary diagnostic information (24), while lymph node biopsy remains essential for confirmation. In our case, treatment-refractory lesions with subsequent lymphadenopathy suggested AITL. Continuous follow-up monitoring is crucial for accurate diagnosis.

In addition, the clinical manifestations of AITL result from hyper-inflammatory reactions, autoimmunity, and immunodeficiency, causing greater susceptibility to infections, leukocytoclastic vasculitis, thyroiditis, arthralgia, and/or arthritis (1). It is even more necessary to be vigilant if there is coexistence of these symptoms and cutaneous involvement. The development of herpes zoster and viral encephalitis during the disease course also supported the diagnosis of AITL.

There has been debate about whether the macular eruptions in AITL represent a coincidental reactive phenomenon or a manifestation of the lymphoma. Although these initial macular eruptions appeared inflammatory, they proved resistant to treatment and progressed from papular lesions to plaques and nodules. Concurrently, histopathological findings evolved from mild perivascular infiltrates with rare cytologically atypical lymphocytes to dense nodular infiltrates with consistent cytological atypia in later stages. This clinicopathologic continuity, despite the pseudoinflammatory presentation of the early eruptions, strongly suggests their lymphomatous nature (7).

## 4 Conclusions

Skin involvement in AITL is often non-specific and easily misdiagnosed as other inflammatory diseases. It is challenging to accurately diagnose AITL in the early stage when only skin manifestations are present without lymphadenopathy. The presented case exemplifies the diagnostic challenges posed by AITL. Given the relatively non-specific clinical and histological features of cutaneous involvement by AITL, clinicopathological correlation is mandatory to establish this diagnosis definitively. Immunohistochemistry for T_FH_ markers and molecular data is sometimes helpful. Continuous observation is necessary in AITL patients who develop skin lesions prior to lymphadenopathy.

## Data Availability

The raw data supporting the conclusions of this article will be made available by the authors, without undue reservation.
