# Supplementary material for: Non-specific skin lesions in angioimmunoblastic T-cell lymphoma lead to diagnosis challenge: a case report and literature review
Source: Front Oncol. 2026 May 14;16:1785153. doi: 10.3389/fonc.2026.1785153 (PMC13215863; doi:10.3389/fonc.2026.1785153)
Supplement: Supplementary file 1 [file DataSheet1.docx]

Supplement Figure


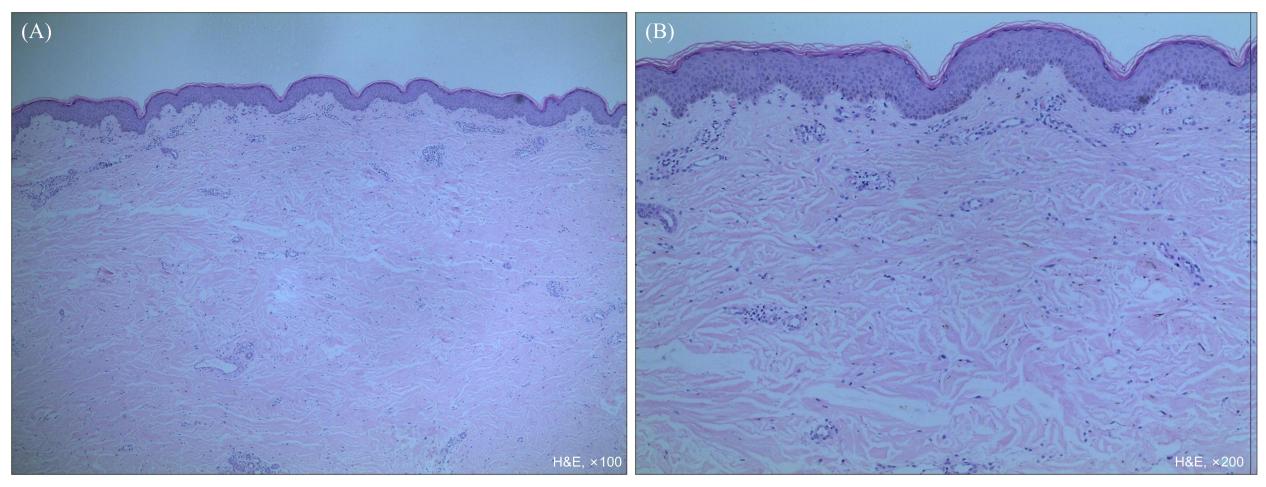
Supplement Figure 1. Skin biopsy of erythema one year ago. The epidermis is essentially normal. There is a mild lymphocytic infiltration around small blood vessels in the upper dermis. Scattered lymphocytes are also present within the lobules of the subcutaneous fat layer.


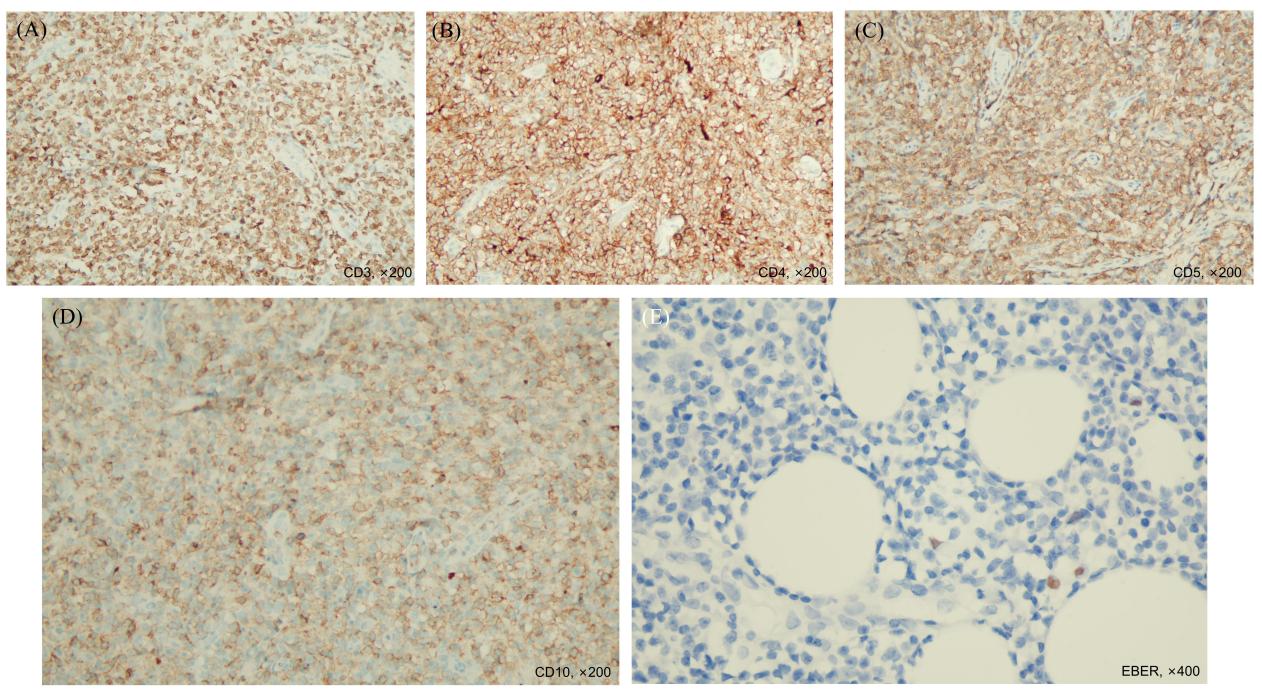


Supplement Figure 2. Immunohistochemistry tests of lymphoid cells in inguinal lymph node biopsy. The lymphoid cells are positive for CD3, CD5, CD4, focal expression of CD10.


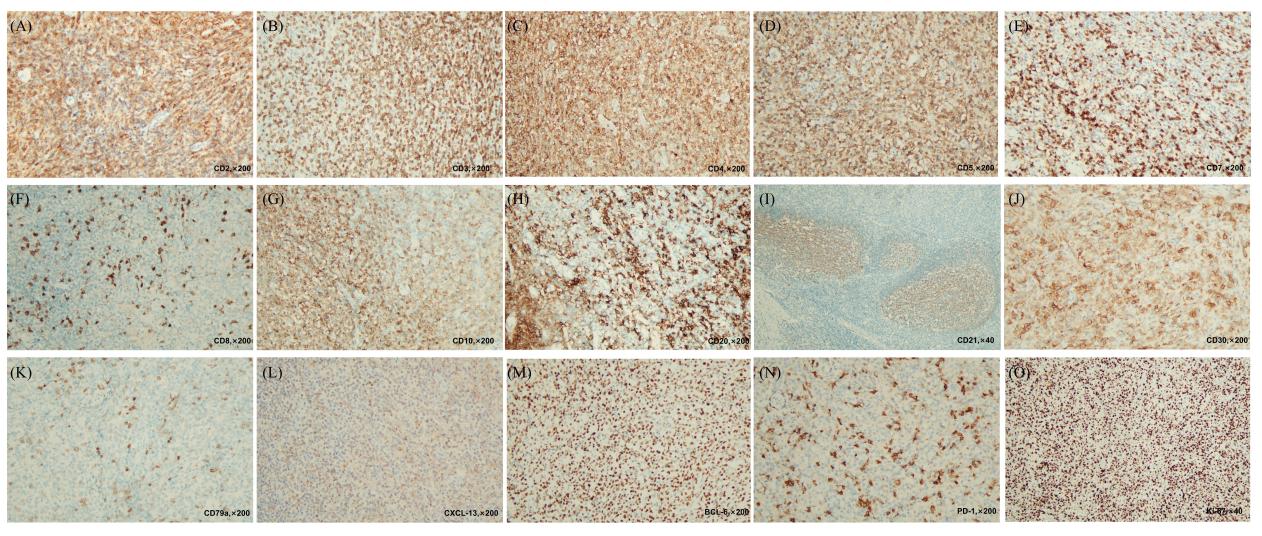


Supplement Figure 3. Immunohistochemistry tests of lymphocytes in inguinal lymph node biopsy. Among lymphocytes, the majority exhibit positivity for CD3, CD5, CD4, CD2, CD7, CD20 and CD79a are only expressed in a minority of cells. CD30 is positive in a subset of transformed cells, and CD21 highlights follicular dendritic cell (FDC) networks. Partial positivity is observed for CD10, BCL-6, CXCL-13, and PD-1, while CD8 is positive in a minority of cells. In situ hybridization demonstrates scattered positivity for EBER. The Ki-67 proliferation index reached 60%–70%. BCL-6, B-cell lymphoma-6; CXCL-13, CXC motif chemokine 13; PD-1, programmed cell death protein 1; EBER-ISH, Epstein-Barr virus (EBV) by in situ hybridization.
